# Bioconversion of rice straw agro-residues by *Lentinula edodes* and evaluation of non-volatile taste compounds in mushrooms

**DOI:** 10.1038/s41598-020-58778-x

**Published:** 2020-02-04

**Authors:** Shuangshuang Gao, Zhicheng Huang, Xi Feng, Yinbing Bian, Wen Huang, Ying Liu

**Affiliations:** 1 College of Food Science and Technology, Huazhong Agricultural University, Wuhan, Hubei 430070 China; 2 Institute of Applied Mycology, Plant Science and Technology College, Huazhong Agricultural University, Wuhan, Hubei 430070 China; 3 Department of Nutrition, Food Science and Packaging, California State University, San Jose, CA 95192 United States

**Keywords:** Biochemistry, Biotechnology

## Abstract

Rice straw was substituted for sawdust at five different ratios of 0, 20%, 40%, 60%, and 80% (Control, RS20, RS40, RS60 and RS80, respectively) to obtain five kinds of *Lentinula edodes*. The effects of adding cropped rice straw to substrate formulas on the proximate composition and non-volatile taste compounds in mushrooms were investigated. The control group had the highest level of MY and BE among the five formulations. The protein levels in mushrooms decreased with the addition of rice straw and the ash levels increased. We found that trehalose, mannitol, and arabitol were the main soluble sugars in the five kinds of mushrooms. The contents of total free amino acids varied from 16.29 to 24.59 mg/g and the highest level of free amino acids was found in mushrooms cultivated from RS20 and RS40. Moreover, the addition of rice straw improved the contents of monosodium glutamate (MSG)-like amino acids in mushrooms. The 5′-Nucleotide levels ranged from 1.66 to 4.48 mg/g and equivalent umami concentration (EUC) value increased with the addition of rice straw. Our results suggest that rice straw is a potential substitute for sawdust to cultivate *L. edodes* with more non-volatile taste compounds.

## Introduction

The white rot basidiomycete (Berk.) Pegler, commonly termed as *Lentinula edodes* or shiitake mushroom, is highly popular with consumers for its great medicine properties and unique flavor in some Asian countries^1–3^. The desirable flavor of the shiitake mushroom consists of volatile and non-volatile components. The former is the basis for the special aroma of *L. edodes*, while the latter includes soluble sugars, organic acids, free amino acids, and 5′-nucleotides, which are responsible for a variety of tastes.

Protein-rich mushrooms can be produced from many lignocellulosic substrates^4^. One of the most popular basal substrates for *L. edodes* production is sawdust. Previous reports have shown that the amount of sawdust in the prescription ranged from 45% to 79%^5,6^. The decreasing forest area is one of the ecological problems all over the world^7^. In order to alleviate the contradiction between the growing shortage of forestry resources and the increasing demand of people in wood products, rice straw, wheat straw, barley straw, vineyard pruning, and hazelnut husk have been used to cultivate *L. edodes*^8–12^. Rice straw is highly available in the world, with about 731 million tons being produced in Africa, Asia, Europe and America every year^13^. In China, the largest agricultural by-product is rice straw, amounting to 184 million tons in 2009^14^. Therefore, evaluation of the use of rice straw for cultivating *L. edodes* is of a great significance for the sustainable development of the forestry resource.

As reported by Zhang, Venkitasamy, Pan, & Wang^15^, in edible mushrooms, the umami ingredients can be affected by several factors, such as species type, part of mushroom, maturity stage, quality grade, processing methods and storage time. To date, the effects of different rice straw/sawdust combinations on the chemical composition of shiitake mushroom, especially non-volatile taste components, are poorly understood.

Therefore, this study also aimed to (1) investigate the effects of different rice straw/sawdust combinations on main tasty components and proximate composition of *L. edodes*, (2) cultivate stronger umami-taste mushroom to meet eating habits in Asia where strong flavors are prized, (3) test the feasibility of rice straw for its application in mushroom production. These results would facilitate the substitution of rice straw for sawdust in mushroom production and provide useful information for improving the non-volatile components in mushrooms.

## Material and Methods

### Mushroom cultivation

The *L. edodes* strain WX1 (ACCC 50926) was cultivated in Mushroom Science and Education Center, Huazhong Agricultural University, Wuhan, China. Five mushroom cultivation substrates were prepared as follows: Control (80% oak sawdust; wheat brane 18%; lime 1%; saccharose 1%), RS20 (20% rice straw; 60% oak sawdust; wheat brane 18%; lime 1%; saccharose 1%), RS40 (40% rice straw; 40% oak sawdust; wheat brane 18%; lime 1%; saccharose 1%), RS60 (60% rice straw; 20% oak sawdust; wheat brane 18%; lime 1%; saccharose 1%), RS80 (80% rice straw; wheat brane 18%; lime 1%; saccharose 1%). The mixture of rice straw and sawdust were wetted for 12–16 h, and then supplemented with 18% wheat brane, 1% lime and 1% saccharose. Based on their dry weight (w/w), all materials were mixed, placed in polypropylene bags (15 × 30 cm) and sterilized at 121 °C for 3.5 h (40 replicates per formulation). After cooling to room temperature, the bags were inoculated along their central vertical axis.

The mushroom house was managed as described by Gong, Xu, Xiao, Zhou, & Bian^5^. Mushroom samples were collected every day from the culture substrates, with the veil being broken and the gill being fully exposed. The biological efficiency (BE) was estimated by the equation: (mushroom fresh weight/substrate dry weight) × 100. Mushroom yield (MY) was recorded for only one flush as follows: mushroom fresh weight/substrate fresh weight. For proximate composition and non-volatile components analysis, the whole fresh *L. edodes* were freeze-dried and then crushed into a powder (80 mesh).

### Proximate composition analysis of fruiting body

The analyses consisting of ash, total nitrogen, and fat were carried out through AOAC (1995) procedures^16^. The total carbohydrate content was estimated as follows: carbohydrates = 100 **−** (ash + fat + protein). The correlation factor 4.38 was used to obtain the total protein from the total nitrogen content.

### Assay of soluble sugars and polyols

The extraction of polyols and soluble sugars from the samples was performed as previously described^17^. The Agilent 1100 high performance liquid chromatography (HPLC) system consisted of a Hi-Plex H column (7.7 × 300 mm, 8 μm, Agilent) and a refractive index detector (RID). Main souble sugars in samples were completely separated in 25 minutes with isocratic elution. The column was operated at 65 **°C**, using deionized water as the mobile phase at a flow rate of 0.6 mL/min, with an injection volume of 5 μL. Each product was compared with the authentic sample (Aladdin, Shanghai) and quantified by the authentic compound calibration curve.

### Assay of organic acids

The extraction and analysis of organic acids were performed as reported by Chen *et al*.^18^. The HPLC system consisted of an InertSustain AQ-C18 column (4.6 × 250 mm, 5 μm) (Shimadzu, Shanghai, China). Authentic standards were used to identify and quantify each organic acid according to the retention time (Sinopharm Chemical Reagent Co. Ltd, Shanghai, China). Each sample was further quantified by comparing its peak area with the related standard compound calibration curve.

### Assay of free amino acids

The extraction and analysis of free amino acids were performed as reported by Chen *et al*.^18^. The free amino acids were analyzed by loading the filtrate onto an L-8900 high-speed amino acid analyzer (Hitachi High-Tech. Corp., Japan).

### Assay of 5′-nucleotides

The extraction and analysis of 5′-Nucleotides were conducted as reported by Chen *et al*.^18^. The HPLC system consisted of an InertSustain AQ-C18 column (4.6 × 250 mm, 5 μm) (Shimadzu, Shanghai, China). 5′-nucleotide standards were used to identify and quantify each 5′-nucleotide (Sigma, U.S.A.).

### Equivalent umami concentration

The equivalent umami concentration [mg MSG per 100 g] represents the monosodium glutamate (MSG) concentration equivalent to the umami intensity given by the mixture of MSG-like amino acids and the flavor 5′-nucleotides (Yamaguchi, Yoshikawa, Ikeda, & Ninomiya)^19^.

### Data analysis

All data were analyzed using ANOVA (analysis of variance) with IBM SPSS Statistics 20. Results were shown as mean ± standard deviation using Duncan’s test (*P* < 0.05). Correlation coefficients (R) between rice straw content in medium and chemical constituents of *L. edodes* fruiting body were computed using commercial software (IBM SPSS Statistics 20, SPSS, Chicago, Illinois, USA).

## Results and Discussion

### Mushroom yield

Spawn run time, yield and BE are shown in Table 1. Artificial logs produced using rice straw-containing substrates were too soft in consistency to be cultivated compared with Control group, and one crop was obtained with all five formulas. After a fifty-day incubation period, five formulas all started to fruit. MY varied between 155.08 and 202.03 g/kg substrate with the highest yield in the Control group. For *L. edodes* cultivation, Morais, Ramos, Matos, & Oliveira^20^ used rice straw to cultivate shiitake but failed to produce any fruiting bodies. Yang *et al*.^12^ determined the effects of substituting different amounts of rice straw (10%, 20%, 30% and 40%) for sawdust in a conventional cultivation substrate formula consisting of 80% oak sawdust, 18.8% bran, 1% gypsum and 0.2% lime and successfully obtained shiitake fruiting bodies. The BE values fluctuated between 36.09% and 49.66%. In our study, the BE value of RS60 is slightly higher than that of Control group. The BE values reported in this study were lower than those obtained by Royse & Sanchez (80.4–98.9%)^21^, but similar to those obtained by Yang *et al*. (36.4–56.6%)^12^ and Gaitán-Hernández & Mata (24.8–55.6%)^10^. Based on the method and substrate used in this study, our BE values were acceptable relative to those mentioned above. Our work shows that a considerable yield and BE can be obtained by replacing up to 80% of oak sawdust with chopped rice straw. Since the rice straw can be available in the cultivation of *L. edodes*, and therefore could pilot a so-called white agricultural revolution in the world.

**Table 1:** Important agronomic traits of every formula in *L. edodes*.

	Control	RS20	RS40	RS60	RS80
Spawn run time (day)	49.87 ± 4.22 a	49.83 ± 4.43 a	50.53 ± 2.98 a	49.10 ± 2.43 a	49.57 ± 2.46 a
MY (g/kg substrate)^a^	202.03 ± 17.40 a	190.42 ± 8.66 ab	155.08 ± 13.94 d	181.20 ± 9.45 bc	164.61 ± 6.94 cd
BE (%)^b^	48.68 ± 4.19 a	36.09 ± 1.64 b	36.61 ± 3.29 b	49.66 ± 2.59 a	39.94 ± 1.68 b

Values (mg/g dry weight) are the means ± SD (n = 3). Means with different letters with a row are significantly different (*P* < 0.05).

MY: mushroom yield calculated as the ratio of mushroom fresh weight/substrate fresh weight.

BE: biological efficiency calculated as the ratio of mushroom fresh weight/substrate dry weight) × 100.

### Proximate composition

**Figure 1:**
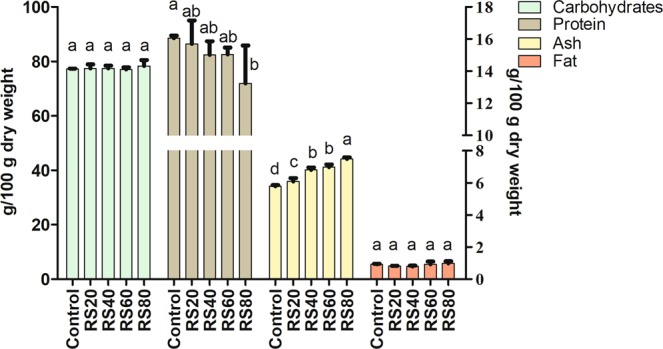
Nutritional value of *L. edodes* harvested in different culture substrates.

In Fig. 1, *L. edodes* fruiting bodies were shown to have a high content of protein and a low content of fat. The decrease of protein levels and increase of ash levels were resulted from the supplementation of rice straw to substrate. Fat and carbohydrates in *L. edodes* of the five groups showed no significant difference (*P* < 0.05). Based on the study by Manzi, Gambelli, Marconi, Vivanti, & Pizzoferrato^22^, the protein (15.19%) and ash (7.08%) contents showed proximate levels to the sample from RS60. Similar findings were obtained in *Pleurotus sapidus* as follows: the mushroom grown on paddy straw mixed with vegetable wastes had a higher protein content in the fruiting bodies than that grown on paddy straw alone^23^. Shiitake reported by Carneiro *et al*.^24^ showed lower contents of proteins (12.76%) and ash (4.29%), but higher contents of fat (1.01%) and Carbohydrates (81.94%). Overall, the nutrition value results have shown that rice straw can decrease protein and increase ash without significant effect on fat and carbohydrate contents of *L. edodes*.

### Soluble sugars and polyols

The perceived sweet taste of certain species can be enhanced by soluble sugars/polyols present in mushrooms. Table 2 shows the contents of polyols and soluble sugars in various mushroom samples from the five different substrates. Mannitol, trehalose and arabitol are reported to be the main soluble sugars or polyols in mushrooms^18,25^ and they were found to be dominant in the five mushroom samples. Their total levels ranged from 177.18 mg/g in the RS20 group to 203.08 mg/g in the RS80 group. The total soluble sugar/polyol concentrations (177.18–203.08 mg/g) of *L. edodes* in the present study are obviously higher than the 78.65–126.1 mg/g obtained by Chen *et al*.^18^, 82.98–127.30 mg/g by Li *et al*.^25^, 169.80 mg/g by Naknaen, Itthisoponkul, & Charoenthaikij^26^, and 141.55–152 mg/g by Yang, Lin, & Mau^27^. The different results are most likely due to the varied mushroom strains and cultivation substrates^27–29^.

**Table 2:** Soluble sugars and polyols of *L. edodes* harvested in different culture substrate.

Sugar/polyols	Control	RS20	RS40	RS60	RS80
Trehalose	73.70 ± 0.74 a	62.92 ± 2.47 c	65.43 ± 0.59 b	40.16 ± 0.61 d	37.14 ± 0.37 e
Mannitol	59.27 ± 0.28 d	60.02 ± 1.74 d	62.26 ± 0.17 c	72.49 ± 1.50 b	79.43 ± 0.55 a
Arabitol	51.45 ± 0.55 e	54.25 ± 1.97 d	63.40 ± 0.35 c	67.13 ± 1.29 b	86.51 ± 0.72 a
Total sugar	184.42 ± 1.00 c	177.18 ± 5.82 d	191.09 ± 0.52 b	179.78 ± 3.38 cd	203.08 ± 1.50 a

Values (mg/g dry weight) are the means ± SD (n = 3). Means with different letters with a row are significantly different (*P* < 0.05).

Generally, the content of trehalose decreased with the addition of rice straw. The content of trehalose of the control group (73.70 mg/g) was about 2.0 times higher than that of RS80 group (37.14 mg/g). However, both mannitol and arabitol increased with the addition of rice straw in culture substrates. The contents of arabitol and mannitol in RS80 group were 1.7 and 1.3 times higher than those of control group, respectively.

### Organic acids

In Table 3, the total level of organic acids was shown to range from 41.67 mg/g to 64.76 mg/g in the five kinds of mushrooms. The total organic acid level (41.67–64.76 mg/g) for the freeze-dried *L. edodes* in this study was obviously lower than 85.40–374.7 mg/g reported by Chen *et al*.^18^ and 121.69–260.00 mg/g by Li *et al*.^25^. Additionally, the contents of total organic acids in the groups of RS40, RS20 and control were higher than those of RS80 and RS60 groups. Furthermore, the main organic acids in RS80 and RS60 groups were found to be malic, acetic and fumaric acids, in contrast to the major organic acids of acetic, malic and succinic in RS40, RS20 and control group. Similar to the total organic acid level, RS80 and RS60 groups were lower than RS40, RS20 and control group in the level of acetic, succinic and tartaric acids. The succinic acid level was found to decrease with addition of rice straw in culture substrates, and it was 9.0-fold higher in the control group than in the RS80 group.

**Table 3:** Contents of organic acids of *L. edodes* harvested in different culture substrate.

Organic acids	Control	RS20	RS40	RS60	RS80
Tartaric acid	2.79 ± 0.07 a	2.78 ± 0.05 a	2.69 ± 0.17 a	2.00 ± 0.12 b	1.97 ± 0.25 b
Malic acid	18.81 ± 0.32 cd	18.35 ± 0.82 d	19.50 ± 0.24 c	23.75 ± 0.84 a	21.74 ± 0.37 b
Ascorbic acid	1.80 ± 0.40 a	1.89 ± 0.16 a	1.26 ± 0.03 b	0.98 ± 0.04 bc	0.81 ± 0.02 c
Acetic acid	20.64 ± 4.91 a	22.09 ± 2.12 a	25.19 ± 0.52 a	8.79 ± 1.78 b	5.91 ± 1.04 b
Citric acid	2.26 ± 0.32 b	2.90 ± 0.63 ab	2.97 ± 0.17 ab	3.48 ± 0.56 a	2.73 ± 0.27 ab
Fumaric acid	4.23 ± 0.02 d	3.62 ± 0.02 e	5.20 ± 0.04 c	8.23 ± 0.30 a	7.26 ± 0.07 b
Succinic acid	11.31 ± 2.65 a	8.81 ± 3.10 a	7.95 ± 3.20 a	1.86 ± 0.32 b	1.25 ± 0.05 b
Total	61.84 ± 7.66 a	60.43 ± 5.98 a	64.76 ± 3.95 a	49.09 ± 2.93 b	41.67 ± 0.48 b

Values (mg/g dry weight) are the means ± SD (n = 3). Means with different letters within a row are significantly different (*P* < 0.05).

In addition, the five kinds of mushrooms had the lowest level of ascorbic acid among the detected organic acids, which accorded with the result reported by Chen *et al*.^18^. Our results revealed malic acid as the main organic acid in both RS80 and RS60 groups, whereas acetic acid was the main organic acid in RS40, RS20 and control groups. However, previous studies have identified succinic acid as the main organic acid in *L. edodes* at different growth stages (Chen *et al*.^18^). Yang, Gu, Liu, Zhou, & Zhang^30^ have reported citric acid as the main organic acid in *L. edodes* with a content of 24.45 mg/g. Our results indicated that rice straw as a substitute for sawdust influenced the organic acids compounds in cultivated *L. edodes*.

### Free amino acids

In Table 4, it can be seen that the content of total free amino acids ranged from 16.29 mg/g in RS60 group to 24.59 mg/g in RS20 group. A total of seven essential amino acids were identified in the five kinds of mushrooms, but tryptophan failed to be detected in all the samples. The essential amino acids showed a similar diversification trend with that of total amino acids. In the five kinds of mushrooms, the major free amino acids consisted of threonine (4.82–8.08 mg/g), glutamic acid (2.00–3.90 mg/g), alanine (0.86–3.38 mg/g) and ornithine (1.07–3.93 mg/g). Additionally, the biologically active compound of γ-aminobutyric acid (GABA) was detected in each of the five groups, with the highest content (1.26 mg/g) in RS20 group, which was higher than 0.35 mg/g (Chen *et al*.) and lower than 2.74 mg/g^31^. Our results suggested that RS20 group could serve as a potential culture substrate to cultivate GABA-rich *L. edodes*.

**Table 4:** Amino acids of *L. edodes* harvested in different culture substrate.

Amino acids	Control	RS20	RS40	RS60	RS80
Aspartic acid	0.08 ± 0.01 d	0.10 ± 0.02 d	0.45 ± 0.07 c	2.27 ± 0.12 a	1.69 ± 0.06 b
Threonine	6.09 ± 1.00 b	7.83 ± 0.83 a	8.08 ± 0.30 a	4.82 ± 0.27 c	5.90 ± 0.18 bc
Serine	0.24 ± 0.03 bc	0.27 ± 0.02 a	0.26 ± 0.00 ab	0.29 ± 0.01 a	0.23 ± 0.01 c
Glutamic acid	2.00 ± 0.29 d	2.76 ± 0.29 c	3.16 ± 0.21 b	3.90 ± 0.13 a	3.43 ± 0.09 b
Glycine	0.50 ± 0.06 c	0.68 ± 0.01 a	0.68 ± 0.01 a	0.56 ± 0.02 b	0.54 ± 0.02 bc
Alanine	2.63 ± 0.38 b	3.38 ± 0.02 a	2.60 ± 0.04 b	0.86 ± 0.03 c	0.87 ± 0.03 c
Cystine	0.68 ± 0.02 ab	0.71 ± 0.03 a	0.63 ± 0.07 b	0.60 ± 0.02 bc	0.54 ± 0.04 c
Valine	0.49 ± 0.07 a	0.55 ± 0.01 a	0.54 ± 0.01 a	0.41 ± 0.01 b	0.38 ± 0.01 b
Methionine	0.07 ± 0.01 a	0.06 ± 0.01 ab	0.07 ± 0.01 a	0.07 ± 0.02 a	0.05 ± 0.01 b
Isoleucine	0.21 ± 0.03 a	0.22 ± 0.01 a	0.20 ± 0.01 a	0.15 ± 0.01 b	0.13 ± 0.00 b
Leucine	0.36 ± 0.03 a	0.35 ± 0.03 a	0.33 ± 0.01 a	0.24 ± 0.01 b	0.23 ± 0.01 b
Tyrosine	0.14 ± 0.04 a	0.07 ± 0.02 c	0.08 ± 0.00 bc	0.10 ± 0.01 abc	0.11 ± 0.01 ab
Phenylalanine	0.34 ± 0.19 a	0.30 ± 0.02 a	0.26 ± 0.01 a	0.20 ± 0.02 a	0.21 ± 0.00 a
GABA	0.82 ± 0.19 b	1.26 ± 0.18 a	0.68 ± 0.12 b	0.09 ± 0.01 c	0.09 ± 0.00 c
Ornithine	3.03 ± 0.54 b	3.93 ± 0.30 a	2.82 ± 0.18 b	1.07 ± 0.04 d	1.90 ± 0.05 c
Lysine	0.31 ± 0.04 a	0.28 ± 0.01 a	0.30 ± 0.01 a	0.21 ± 0.01 b	0.21 ± 0.01 b
Proline	0.19 ± 0.01 a	0.18 ± 0.01 a	0.17 ± 0.01 a	not detected	not detected
Histidine	0.36 ± 0.06 a	0.39 ± 0.01 a	0.35 ± 0.01 a	0.24 ± 0.01 b	0.28 ± 0.01 b
Arginine	0.88 ± 0.11 b	1.24 ± 0.03 a	0.76 ± 0.03 c	0.21 ± 0.02 e	0.34 ± 0.00 d
Essential AA	7.88 ± 1.04 b	9.59 ± 0.78 a	9.79 ± 0.32 a	6.10 ± 0.31 c	7.11 ± 0.21 bc
Total AA	19.43 ± 2.52 b	24.59 ± 0.63 a	22.44 ± 0.29 a	16.29 ± 0.69 c	17.14 ± 0.52 c
MSG-like	2.09 ± 0.30 e	2.87 ± 0.32 d	3.62 ± 0.28 c	6.17 ± 0.24 a	5.12 ± 0.15 b
Sweet	9.64 ± 1.40 b	12.36 ± 0.83 a	11.80 ± 0.26 a	6.53 ± 0.33 c	7.54 ± 0.23 c
Bitter	2.72 ± 0.38 b	3.12 ± 0.06 a	2.52 ± 0.05 b	1.52 ± 0.08 c	1.62 ± 0.04 c
Tasteless	1.95 ± 0.19 b	2.32 ± 0.18 a	1.69 ± 0.15 c	1.00 ± 0.03 d	0.95 ± 0.05 d

Values (mg/g dry weight) are the means ± SD (n = 3). Means with different letters with a row are significantly different (*P* < 0.05).

GABA, γ-aminobutyric acid. Essential amino acids, Thr + Val + Met + Ile + Leu + Phe + Lys + Trp, while Trp was not detected in this study. MSG-like, monosodium glutamate-like, Asp + Glu; Tasteless, Cys + Tyr + Lys + GABA; Sweet, Thr + Ser + Gly + Ala + Pro; Bitter, Val + Met + Ile + Leu + Phe + His + Arg + Trp.

In previous studies, MSG-like, sweet, bitter and tasteless have been determined as the four taste features of free amino acids^27,32^. A comparison analysis of the data indicated that the five kinds of mushrooms had the highest sweet components and the lowest tasteless components. However, Mau, Lin, & Chen^33^ found that the bitter components were dominated in several medicinal mushrooms, including Sung Shan Ling Chih (*Ganoderma tsugae*), Ling Chih (*Ganoderma lucidum*) and Yun Chih (*Coriolus versicolor*). The MSG-like components consisted of aspartic and glutamic acids, which were characterized by the taste of MSG and 5′-nucleotide and thus gave the umami taste or the most typical mushroom taste^34^. The content of MSG-like components was 2.09–6.17 mg/g in our study. Previous studies have divided the contents of MSG-like amino acids into three levels: high (>20 mg/g), middle (5–20 mg/g) and low (<5 mg/g)^27^. In the present study, the content of MSG-like amino acids was at the middle level in the RS80 and RS60 groups (5.12 and 6.17 mg/g, respectively), but at low levels in RS40, RS20 and control groups (3.62, 2.87 and 2.09 mg/g, respectively). Similar to its level in total free amino acids, the RS20 group had the highest contents of sweet amino acids (12.36 mg/g), bitter amino acids (3.12 mg/g) and tasteless amino acids (2.32 mg/g) among the five groups.

### 5′-nucleotides

The 5′-nucleotides in mushrooms have been reported as a potential contributor to the umami taste^19^. Five 5′-nucleotides (5′-AMP, 5′-CMP, 5′-GMP, 5′-IMP and 5′-UMP) were detected in the five kinds of mushrooms (Table 5). The total 5′-nucleotide contents varied from 1.66 mg/g in RS20 group to 4.48 mg/g in RS80 group, indicating the increase of the total 5′-nucleotides with the addition of rice straw. Furthermore, 5′-AMP (1.18–2.78 mg/g) and 5′-CMP (0.16–1.29 mg/g) were found as the main 5′-nucleotides in the five different mushroom samples.

**Table 5:** 5′-Nucleotide levels of *L. edodes* harvested in different culture substrate.

	Control	RS20	RS40	RS60	RS80
5′-CMP	0.40 ± 0.11 b	0.30 ± 0.05 bc	0.22 ± 0.01 bc	0.16 ± 0.08 c	1.29 ± 0.19 a
5′-UMP	0.07 ± 0.00 b	0.05 ± 0.01 b	0.06 ± 0.00 b	0.02 ± 0.01 b	1.04 ± 0.20 a
5′-GMP	0.02 ± 0.01 b	0.02 ± 0.00 b	0.01 ± 0.00 b	0.01 ± 0.01 b	0.72 ± 0.16 a
5′-IMP	0.07 ± 0.01 bc	0.11 ± 0.01 a	0.05 ± 0.00 cd	0.08 ± 0.01 b	0.05 ± 0.02 d
5′-AMP	1.67 ± 0.03 bc	1.18 ± 0.06 d	1.75 ± 0.01 b	2.78 ± 0.02 a	1.37 ± 0.39 cd
Total	2.23 ± 0.13 c	1.66 ± 0.02 d	2.09 ± 0.01 c	3.06 ± 0.12 b	4.48 ± 0.35 a
Flavor 5′-nucleotide	0.09 ± 0.01 b	0.13 ± 0.01 b	0.06 ± 0.00 b	0.10 ± 0.02 b	0.76 ± 0.16 a
EUC value	10.42 ± 1.94 c	12.61 ± 1.50c	15.34 ± 1.16 c	30.93 ± 1.26 b	84.49 ± 11.72 a

Values (mg/g dry weight) are the means ± SD (n = 3). Means with different letters with a row are significantly different (*P* < *0.05*). 5′-AMP, 5′-adenosine monophosphate; 5′-CMP, 5′-cytosine monophosphate; 5′-GMP, 5′-guanosine monophosphate; 5′-UMP, 5′-uridine monophosphate; 5′-IMP, 5′-inosine monophosphate. Flavor 5′-nucleotide, 5′-GMP + 5′-IMP.

Yang, Lin, & Mau^27^ defined flavor 5′-nucleotides in three ranges: high (>5 mg/g), medium (1–5 mg/g) and low (<1 mg/g). In the present study, the content of flavor 5′-nucleotides was 0.09–0.76 mg/g and thus in the low range for all the *L. edodes* samples from the five different substrates. Interestingly, flavor 5′-nucleotides showed a sudden decrease in RS60 when we added sawdust into *L. edodes* cultivation medium, and remained at the same level in RS60, RS40, RS20 and control group (*P* < 0.05). Overall, a high proportion of rice straw in medium might contribute to increase the 5′-nucleotides content in *L. edodes*.

### Equivalent umami concentration

**Figure 2:**
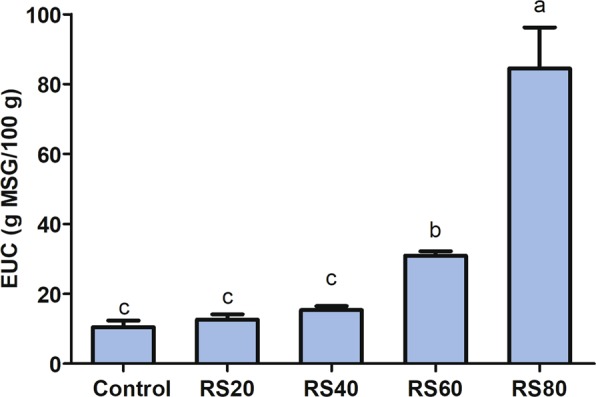
Equivalent umami concentration (EUC) values of *L. edodes* harvested from different culture substrate. Values were expressed as mean ± SD (n = 3).

Figure 2 shows the equivalent umami concentration (EUC) values for the umami taste based on the synergistic effects of MSG-like components and 5′-nucleotides. The results showed that the EUC values of the five kinds of mushrooms ranged from 10.42 to 84.49 g MSG/100 g, which were mostly lower than those reported in earlier studies^25,26,32^. Previous studies defined the EUC values at four levels: 10% (<0.1 g MSG/g), 10%-100% (0.1–1 g MSG/g), 100%-1000% (1–10 g MSG/g), and >1000% (>10 g MSG/g)^35^. Accordingly, the EUC values were at the second level for all samples (10–100 g MSG/100 g). The EUC value of RS80 group was 2.7-, 5.5-, 6.7- and 8.1-fold higher than those of RS60, RS40, RS20 and control group, mainly due to the high level of 5′-GMP in RS80 group. Moreover, the EUC value increased with the addition of rice straw, indicating that rice straw as a substitute for sawdust improved the umami taste components of *L. edodes*.

**Figure 3:**
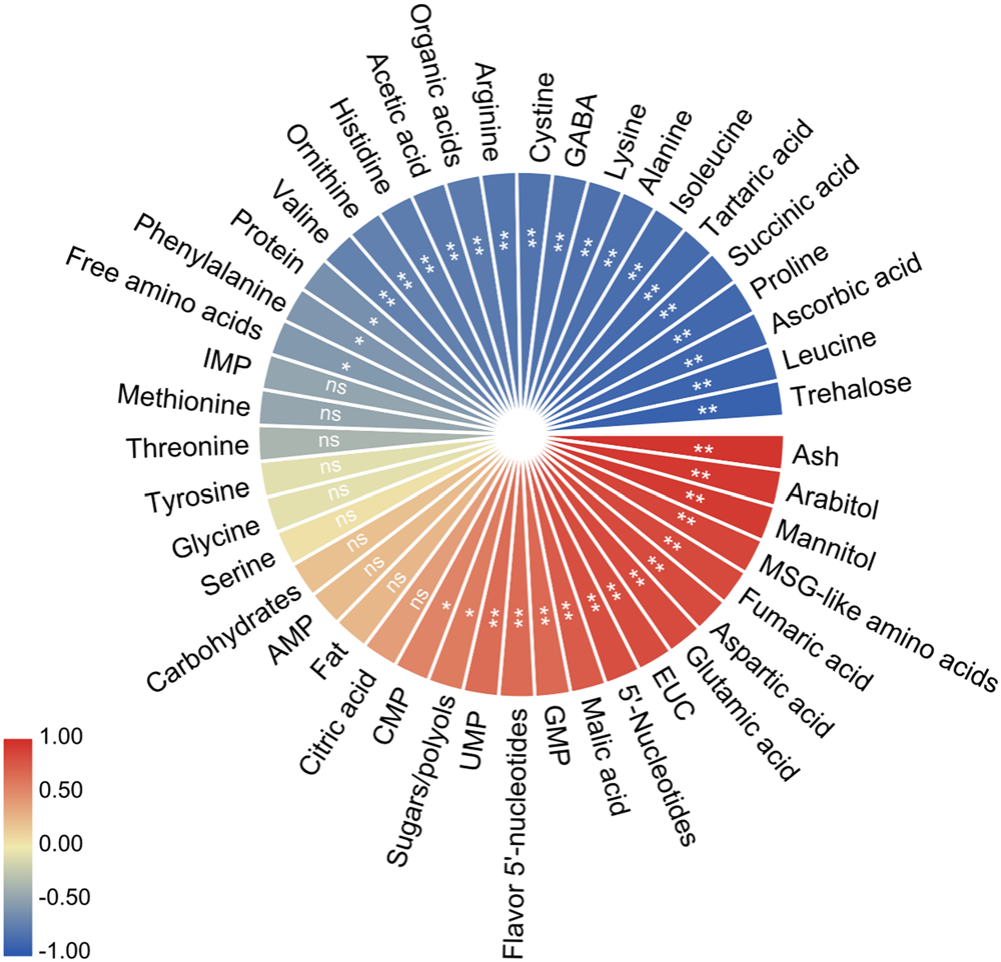
Correlation between rice straw content in culture substrate and chemical constituents of *L. edodes*. ^**^P < 0.01; ^*^P < 0.05; ns, not significantly.

The correlation analysis between rice straw ratios and chemical components is shown in Fig. 3. The results showed that rice straw ratios in substrate had a significant negative relationship with the contents of trehalose, leucine, ascorbic acid, proline, succinic acid, tartaric acid, isoleucine, alanine, lysine, GABA, cystine, arginine, acetic acid, histidine, ornithine, valine, and organic acids in *L. edodes* fruiting bodies (*P* < 0.01). One the contrary, rice straw ratios had a significant positive correlation with the contents of ash, arabitol, mannitol, MSG-like amino acids, fumaric acid, aspartic acid, glutamic acid, malic acid, GMP, UMP, EUC, 5′-nucleotides and flavor 5′-nucleotides (*P* < 0.01). Similar results have also been reported in previous studies, such as the effect of the composition of culture substrates on the nutritional value of mushrooms^23,36^. Gaitán-Hernández, Esqueda, Gutiérrez, & Beltrán-García^37^ have reported that substrate composition is correlated with the growth of *L. edodes*. During the growth, mushrooms would use the most accessible lignocellulosic components of the substrate^38^. The lignocellulose degradation capacity of *L. edodes* was influenced by the culture substrates (such as wheat straw, barley straw, and vineyard pruning)^9^. When grown under different culture substrates, cultures of white-rot basidiomycetes varied largely in the yield of lignocellulolytic enzymes^39,40^. These hydrolytic enzymes were required for the conversion of the major components in substrate (cellulose, hemicellulose and lignin) into low-molecular-weight compounds and their assimilation for mushroom nutrition^41^. We suggested that rice straw as a substitute for sawdust at different ratios would change the lignocellulose composition to affect the non-volatile taste compounds in cultivated *L. edodes*. However, little information can be obtained concerning the relationship between lignocellulosic biomass and non-volatile taste composition. Thus, the effects of lignocellulosic biomass (rice straw or sawdust) on the degradation of lignin and carbohydrates need to be elucidated in future studies.

## Conclusion

In summary, rice straw was substituted for sawdust at five different ratios of 0, 20%, 40%, 60%, and 80% to cultivate *L. edodes* and five kinds of mushrooms were successfully obtained with a considerable mushroom yield and biological efficiency. Adding rice straw into substrates could decrease protein content but increase the EUC values in *L. edodes* (*P* < 0.05). Our results suggested the possibility that up to 80% of oak sawdust can be replaced with chopped rice straw to cultivate *L. edodes* with more non-volatile taste compounds.
